# Supplementary material for: Aromatic residues in mobile regions distal to the active site support the closed conformation of E. coli DXPS
Source: bioRxiv. 2025 Nov 19:2025.10.24.684457. Preprint. [Version 2] doi: 10.1101/2025.10.24.684457 (PMC12633525; doi:10.1101/2025.10.24.684457)
Supplement: 1 [file NIHPP2025.10.24.684457V2-supplement-1.pdf]

bioRxiv preprint doi: https://doi.org/10.1101/2025.10.24.684457; this version posted November 19, 2025. The copyright holder for this preprint (which was not certified by peer review) is the author/funder, who has granted bioRxiv a license to display the preprint in perpetuity. It is made available under aCC-BY 4.0 International license.

Supporting Information

Aromatic residues in mobile regions distal to the active site support the closed conformation of *E. coli* DXPS

Lydia J. Kramer[1], Steven L. Austin[2], Ananya Majumdar[3], Noah D. Smith[1], H. Lee Woodcock[4], and Caren L. Freel Meyers[1*]

[1]Department of Physiology, Pharmacology, and Therapeutics, Johns Hopkins School of Medicine, Baltimore, MD, 21205, United States

[2]Department of Natural Sciences, New College of Florida, Sarasota, FL, 34243, United States

[3]Biomolecular NMR Center, Johns Hopkins University, Baltimore, Maryland, 21218, United States

[4]Department of Chemistry, University of South Florida, Tampa, FL, 33620, United States

*Corresponding author: Caren Freel Meyers, cmeyers@jhmi.edu

Table of Contents

Figure S1: DXPS global conformation……………………………………………………………..S3

Table S1: Variant DXPS primer sequence and annealing temperature for site directed mutagenesis……………………………………………………………………………………S4

Figure S2: Correlation between aromatic cluster pairs and DrDXPS simulation principal components………………………………………………………………………………………S5

Figure S3: Aerobic DXP formation Michaelis Menten Curves……………………………….S6-7

Figure S4: Secondary Structure and apparent melting temperature comparison of WT and variant DXPS…………………………………………………………………………………....S8

Table S2: WT and variant DXPS apparent melting temperature…………………………….S8

Figure S5: Duplicate curves comparing WT and variant DXPS AP and IP ThDP tautomers using steady state CD signatures……………………………………………………………………S9

Figure S6: WT and variant duplicate AP and PLThDP IP signatures………………………S9

Figure S7: PLThDP formation on WT and variant DXPS…………………………………….S10

Figure S8: Trypsinolysis scheme……………………………………………………………….S11

bioRxiv preprint doi: https://doi.org/10.1101/2025.10.24.684457; this version posted November 19, 2025. The copyright holder for this preprint (which was not certified by peer review) is the author/funder, who has granted bioRxiv a license to display the preprint in perpetuity. It is made available under aCC-BY 4.0 International license.

Figure S9: Accumulation of trypsin digest products……………………….……….……………...S12

Figure S10: Raw triplicate trypsin digest gels...…………………………………………………....S13

Figure S11: Duplicate LThDP persistence and clarified solution analysis of WT and variant DXPS using steady state CD……………………………………………………...…...……..…S14-15

Figure S12: WT DXPS and dialysis buffer comparison of AP and LThDP IP tautomers by steady state CD……………………………………………………………………………………………..S15

Figure S13: Aromatic cluster disrupting variants show slower PLThDP at 25 °C……….……..S16

Figure S14: Observation of intermediate ejection from aromatic disrupting variants..………..S17

Figure S15: Comparison of HEThDP and F298L unknown product by NMR……………....S18-19

Figure S16: Discernment between pre- and post-decarboxylation intermediate by NMR…..……………………………………………………………………………………………..S20

Figure S17: Duplicate data comparing H299A to aromatic removing variants…………………S21

References…………………………………………………………………………………………S22

bioRxiv preprint doi: https://doi.org/10.1101/2025.10.24.684457; this version posted November 19, 2025. The copyright holder for this preprint (which was not certified by peer review) is the author/funder, who has granted bioRxiv a license to display the preprint in perpetuity. It is made available under aCC-BY 4.0 International license.

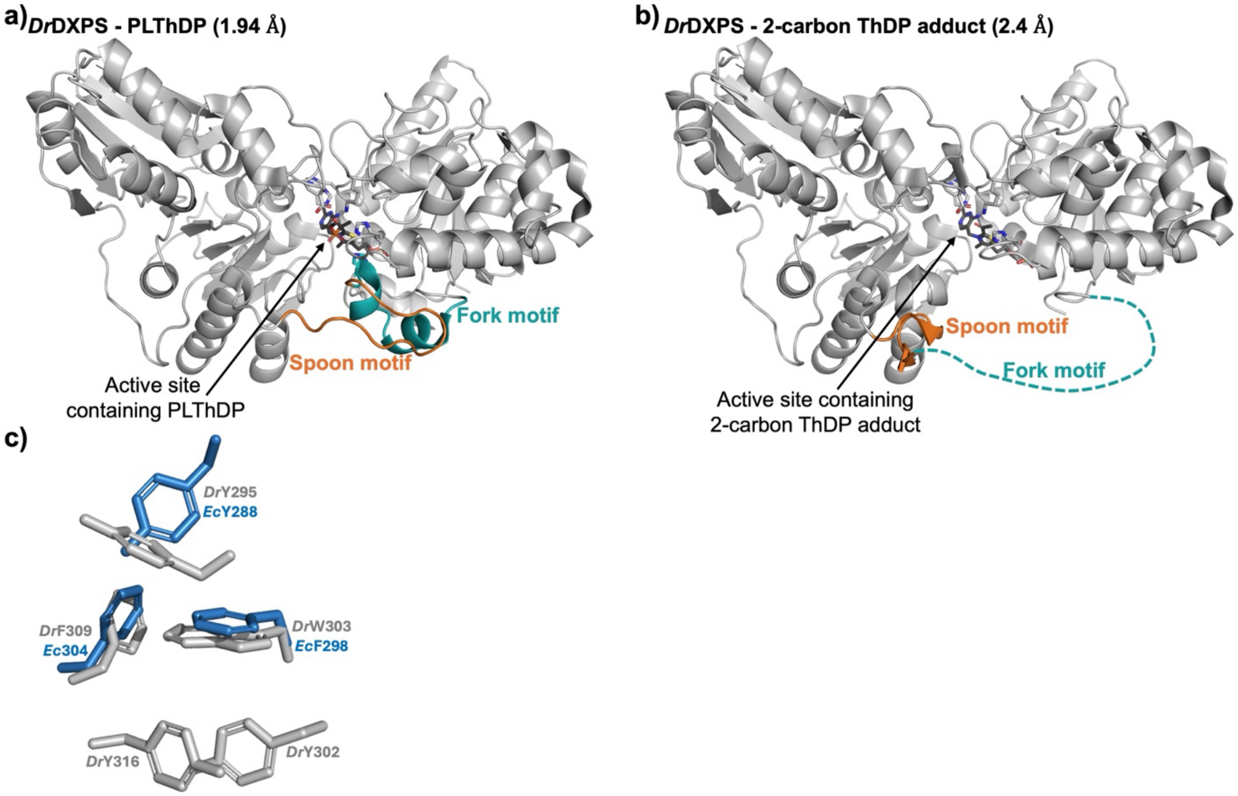

Figure S1: DXPS undergoes global conformational changes during catalysis, two of which have been determined previously: the closed (a, PBDID: 6ouv) and open (b, PBDID: 6ouw).[1] c) A structure alignment of the *Dr* (grey) crystal structure and *Ec* (blue) AlphaFold model reveals spatial alignment of the spoon and fork aromatic residue clusters.

bioRxiv preprint doi: https://doi.org/10.1101/2025.10.24.684457; this version posted November 19, 2025. The copyright holder for this preprint (which was not certified by peer review) is the author/funder, who has granted bioRxiv a license to display the preprint in perpetuity. It is made available under aCC-BY 4.0 International license.

Table S1: Variant DXPS primer sequence and annealing temperature for site directed mutagenesis

Primer	Sequence 5' ⟶ 3'	Ta (°C)
Y288A Forward	CAAAAAAGGTCGTGGT<u>GCT</u>GAACC	69°C
Y288A Reverse	GTCATGATATGCAGGAACTGCGGG	
Y288F Forward	GTGGT<u>TTT</u>GAACCGGCAG	61°C
Y288Q Forward	GTGGT<u>CAA</u>GCGGCAG	55°C
Y288F/Q Reverse	GACCTTTTTTGGTCATGATATGC	
F298A Forward	GATCACT<u>GCC</u>CACGCCGT	61°C
F298L Forward	GATCACT<u>TTG</u>CACGCCGTG	66°C
F298A/L Reverse	GGGTCTTTTTCTGCCGGTTC	
F304A Forward	CCTAAA<u>GCT</u>GATCCCTCCAGCGG	66°C
F304L Forward	CCTAAA<u>TTA</u>GATCCCTCCAGCGG	60°C
F304A/L Reverse	CACGGCGTGGAAAGTGATCGG	

bioRxiv preprint doi: https://doi.org/10.1101/2025.10.24.684457; this version posted November 19, 2025. The copyright holder for this preprint (which was not certified by peer review) is the author/funder, who has granted bioRxiv a license to display the preprint in perpetuity. It is made available under aCC-BY 4.0 International license.

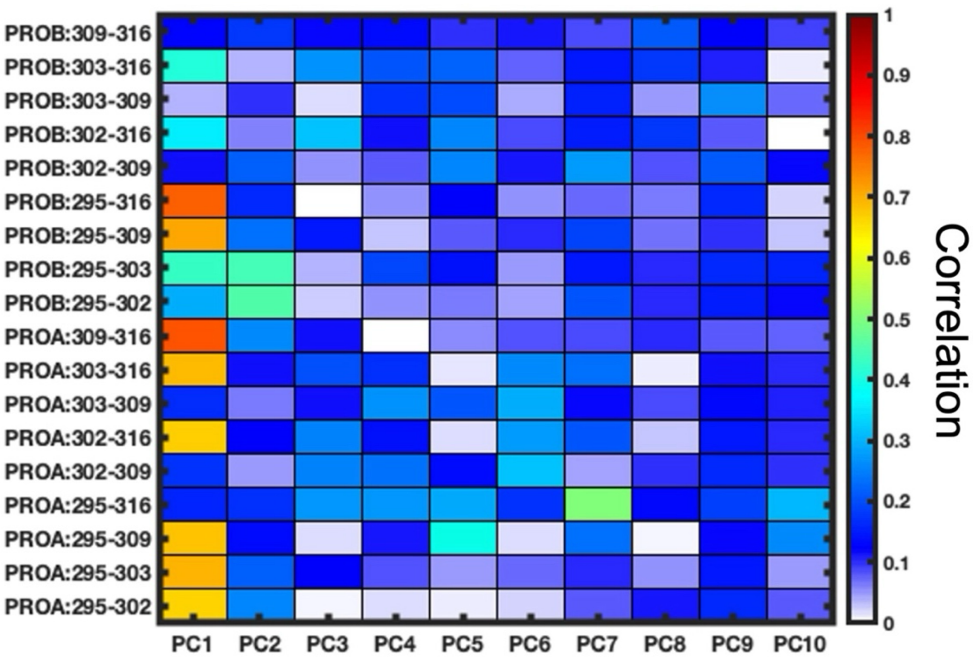

Figure S2: Aromatic cluster dynamics are correlated to global DXPS dynamics. The correlation values were computed between the aromatic cluster pair distances and the principal components.

bioRxiv preprint doi: https://doi.org/10.1101/2025.10.24.684457; this version posted November 19, 2025. The copyright holder for this preprint (which was not certified by peer review) is the author/funder, who has granted bioRxiv a license to display the preprint in perpetuity. It is made available under aCC-BY 4.0 International license.

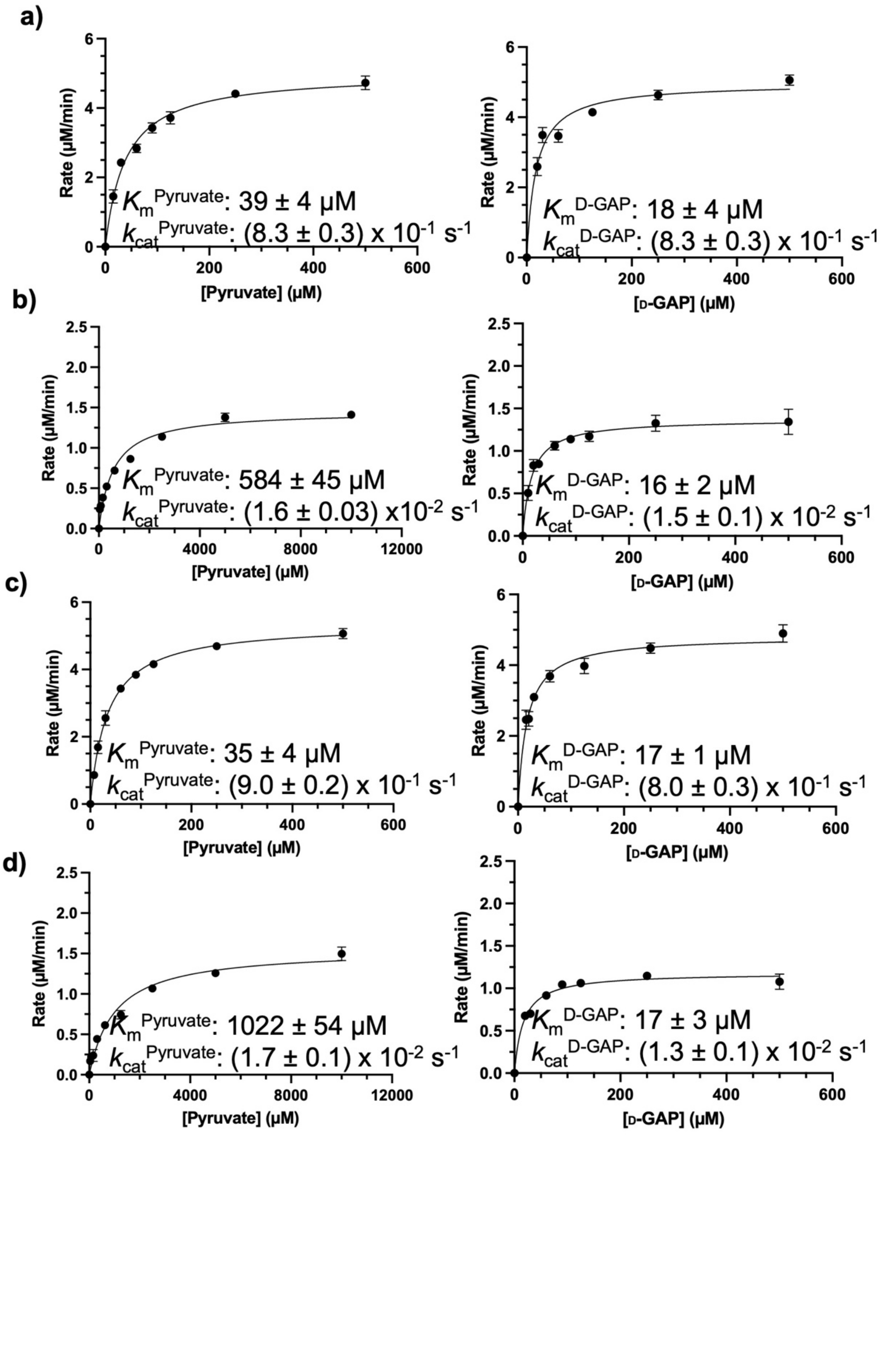

bioRxiv preprint doi: https://doi.org/10.1101/2025.10.24.684457; this version posted November 19, 2025. The copyright holder for this preprint (which was not certified by peer review) is the author/funder, who has granted bioRxiv a license to display the preprint in perpetuity. It is made available under aCC-BY 4.0 International license.

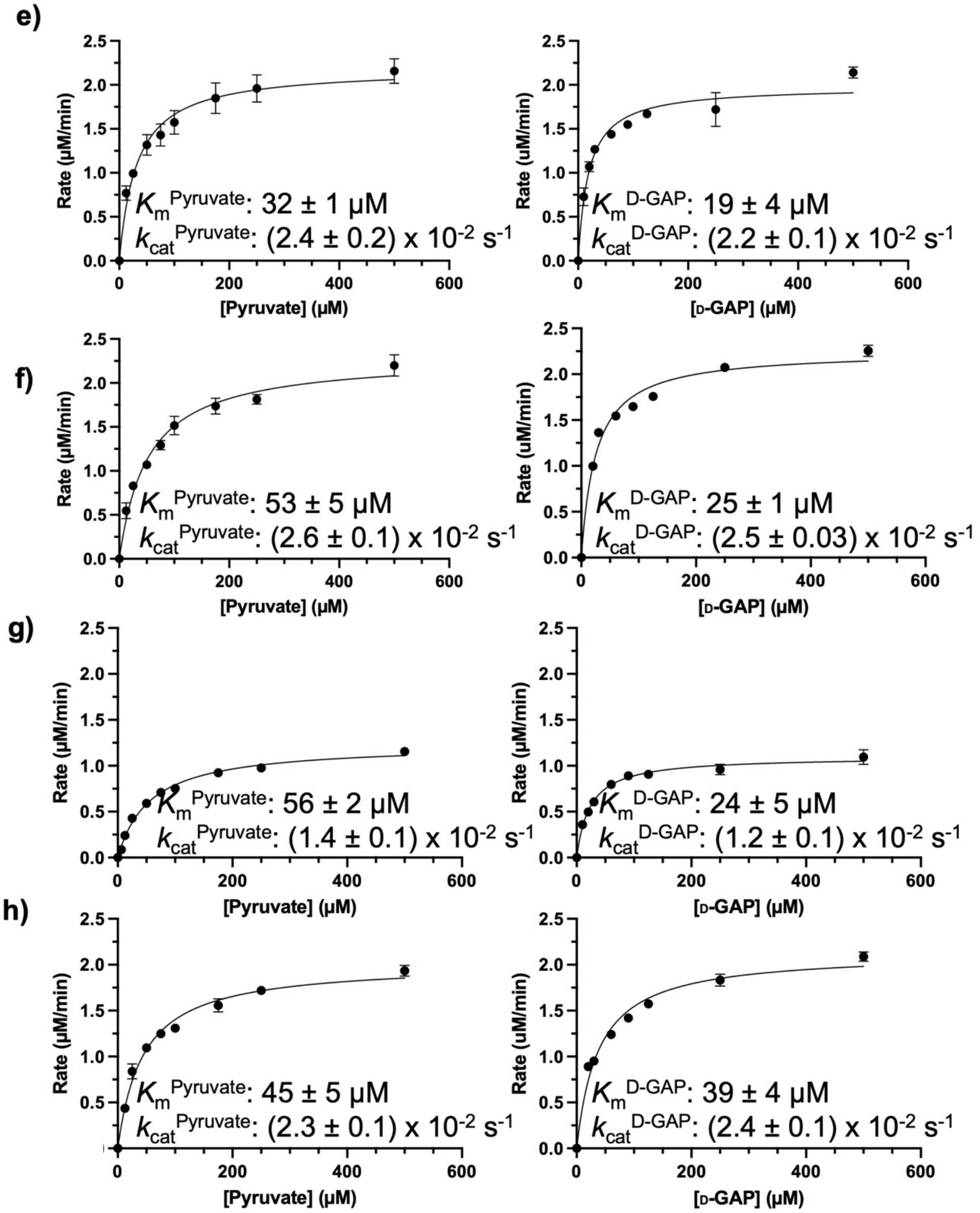

Figure S3: Determination of kinetic parameters for DXP formation on WT and variant DXPS. Substrate concentrations are varied (pyruvate, left; D-GAP, right) under aerobic conditions on WT (a), Y288A (b), Y288F (c), Y288Q (d), F298A (e), F298L (f), F304A (g), and F304L (h). Error bars represent the standard error of the mean. Errors in (K_m) and (k_{cat}) represent standard error determined from 4 experiments.

bioRxiv preprint doi: https://doi.org/10.1101/2025.10.24.684457; this version posted November 19, 2025. The copyright holder for this preprint (which was not certified by peer review) is the author/funder, who has granted bioRxiv a license to display the preprint in perpetuity. It is made available under aCC-BY 4.0 International license.

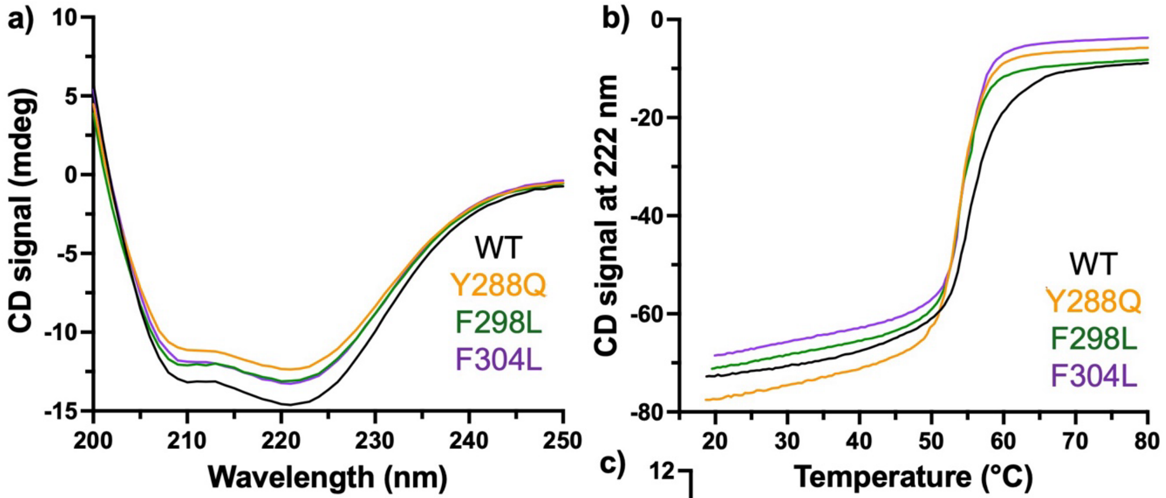

Figure S4: Representative secondary structure (a) and thermal stability (b,c) curves of WT and variant DXPS measured by CD (n = 3). a) The α helical nature is maintained on variant DXPS. b) CD signal was measured from 20–80 °C to determine the thermal stability. c) The first derivative was calculated to estimate the apparent melting temperature (thermal denaturation was not reversible) using the maximum of each curve.

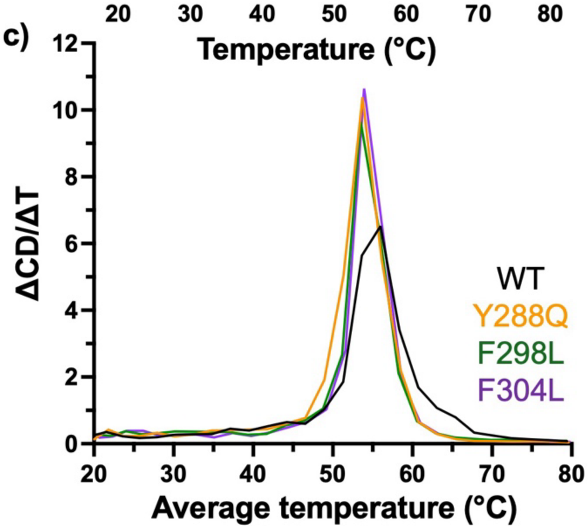

Table S2: WT and variant DXPS apparent melting temperature

***Ec* DXPS**	**$^{app}T_m$ (°C)**
WT	55.0 ± 0.5
Y288Q	54.0 ± 0.5
F298L	53.2 ± 0.4
F304L	54.3 ± 0.2

Error represents standard error, n = 3

bioRxiv preprint doi: https://doi.org/10.1101/2025.10.24.684457; this version posted November 19, 2025. The copyright holder for this preprint (which was not certified by peer review) is the author/funder, who has granted bioRxiv a license to display the preprint in perpetuity. It is made available under aCC-BY 4.0 International license.

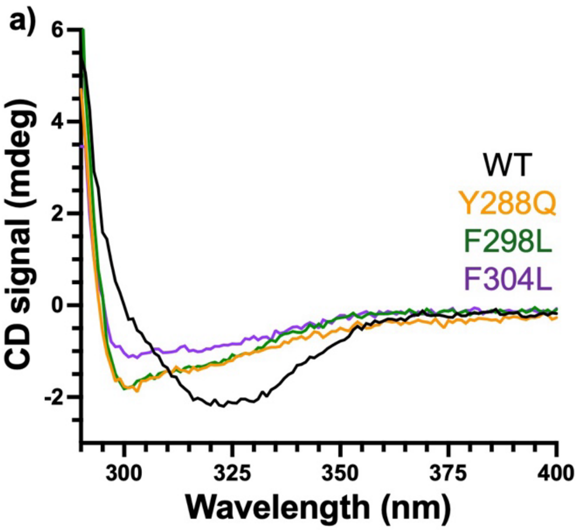

Figure S5: Replicate CD curves of WT and variant DXPS AP (a) and LThDP IP (b) signals. LThDP was formed upon the addition of pyruvate (final concentration of $5\text{x}K_m^{\text{Pyr}}$) in each case.

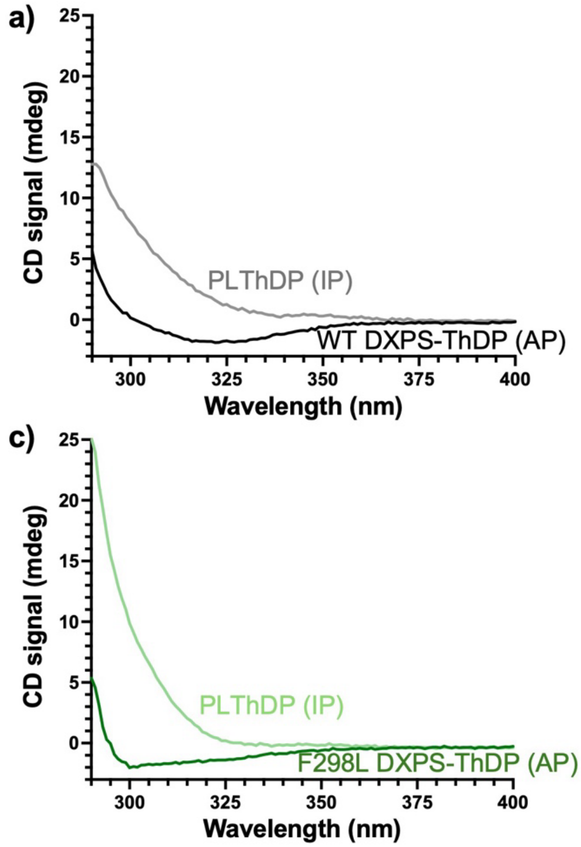

Figure S6: AP and PLThDP signals on WT and DXPS variants. CD scan of WT DXPS (a), Y288Q (b), F298L (c), and F304L (d) bound to ThDP or PLThDP, formed from the addition of 5 mM MAP.

bioRxiv preprint doi: https://doi.org/10.1101/2025.10.24.684457; this version posted November 19, 2025. The copyright holder for this preprint (which was not certified by peer review) is the author/funder, who has granted bioRxiv a license to display the preprint in perpetuity. It is made available under aCC-BY 4.0 International license.

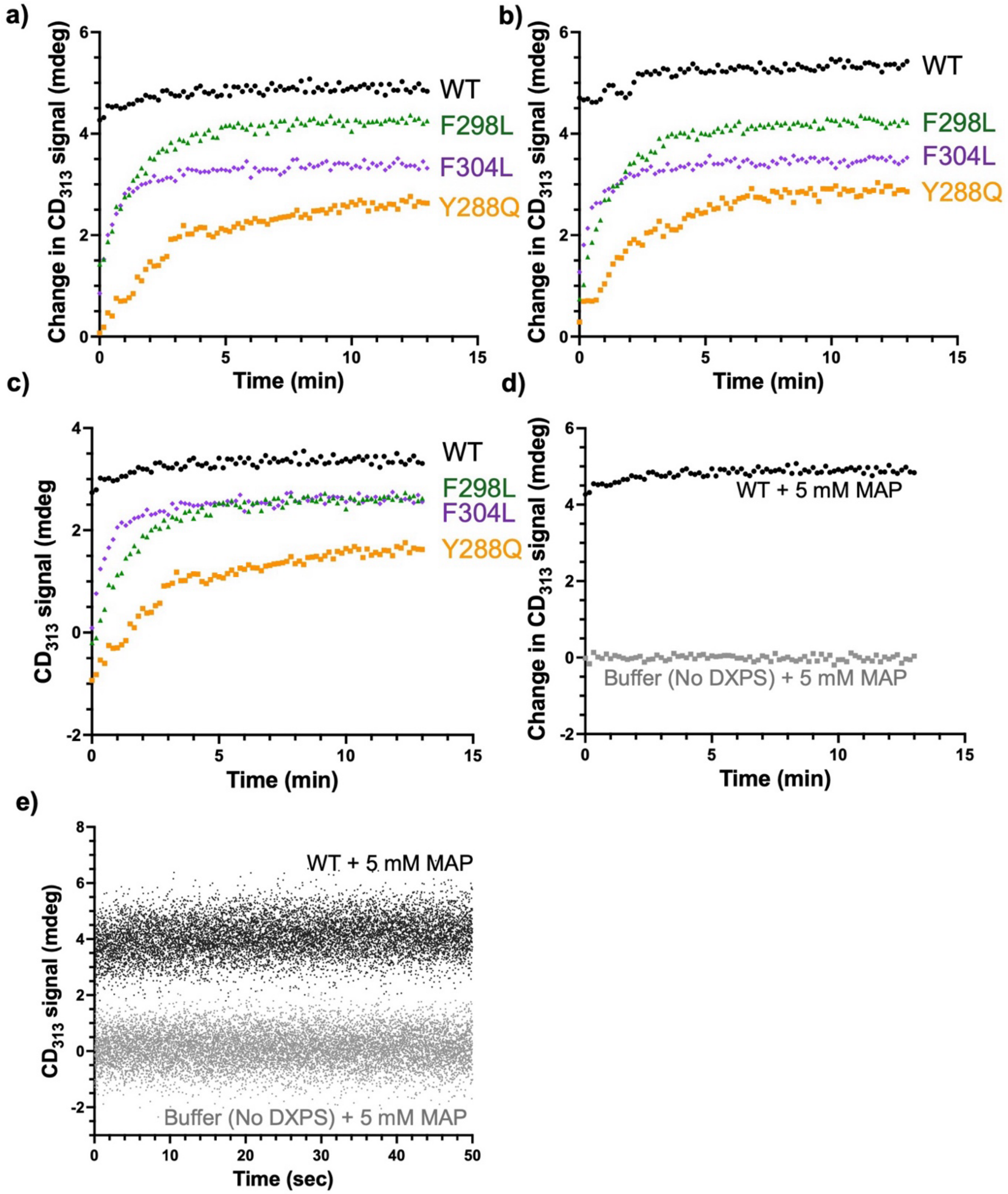

Figure S7: PLThDP formation on WT and DXPS variants. Accumulation of CD_{313} signal from AP signal subtracted, as shown in Figure 5d, (a) and duplicate data (b) observing PLThDP formation by monitoring the change in CD_{313} signal over 13 minutes. c) Unnormalized data from Figures 5d and S7a monitoring PLThDP signal accumulation. (d,e) Comparison of the PLThDP signal observed when 5 mM MAP is added to WT DXPS (black) and or buffer (grey, no DXPS control) in time resolved (d) and normalized, steady-state (e) CD experiments.

bioRxiv preprint doi: https://doi.org/10.1101/2025.10.24.684457; this version posted November 19, 2025. The copyright holder for this preprint (which was not certified by peer review) is the author/funder, who has granted bioRxiv a license to display the preprint in perpetuity. It is made available under aCC-BY 4.0 International license.

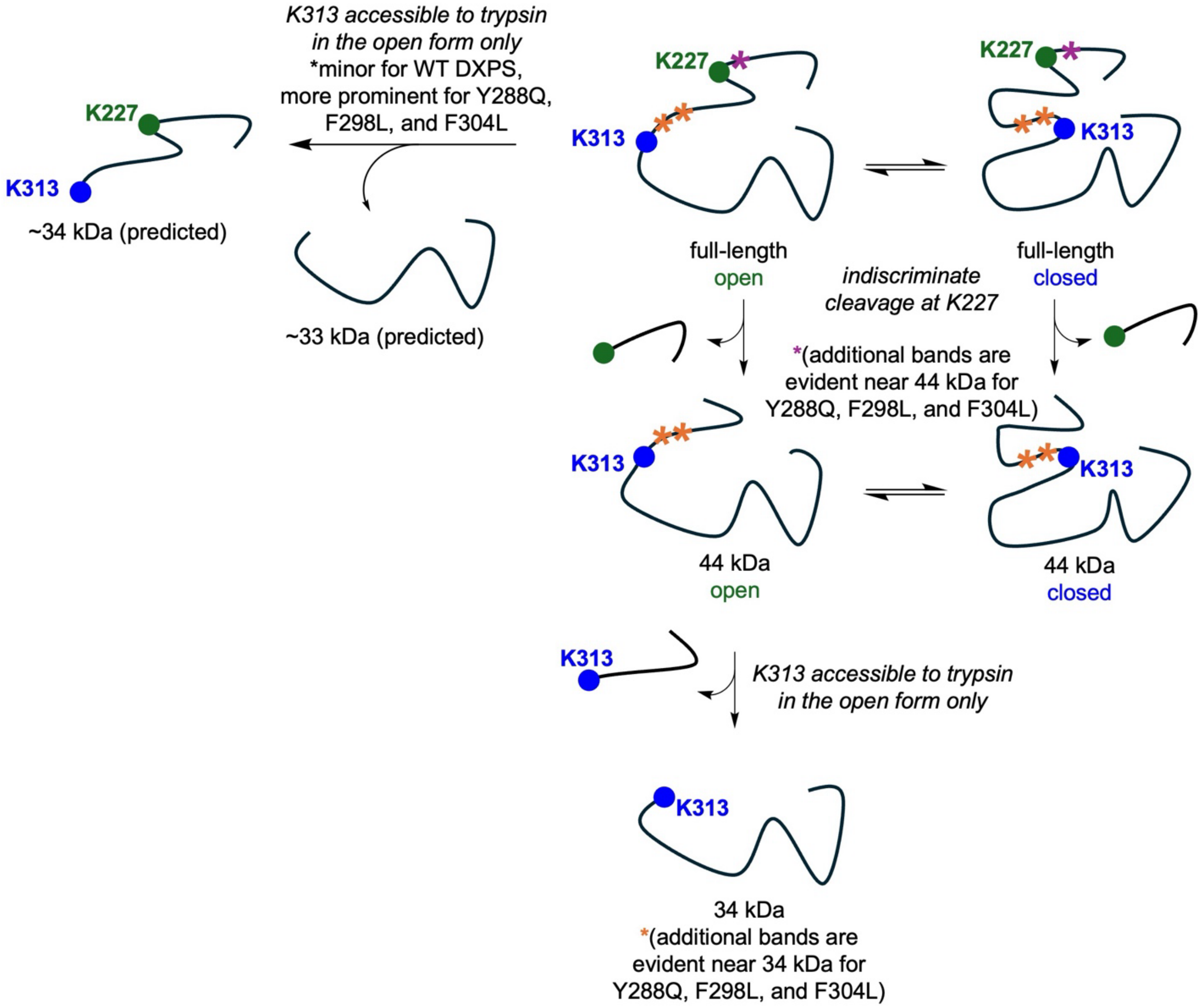

Figure S8: Trypsinolysis captures shifts in conformational equilibria of WT and variant *Ec*DXPS. Two major cleavage products were previously identified on the WT DXPS with accessibility dependent on global enzyme conformation.[2] Cleavage of K227 occurs indiscriminately on either conformation, producing a 44 kDa peptide fragment. However, cleavage of K313 in the fork motif occurs only upon formation of the open conformation where the fork motif is disordered. Y288Q, F298L, and F304L appeared to exist predominately in an open conformation, evidenced by the rapid accumulation of the 34 kDa peptide. Additionally, digestion of each variant led to “doublet” bands near the 34 and 44 kDa major cleavage sites. We reason that a predominately open enzyme may be cleaved at K313 before or after K227, which would produce 34 and 33 kDa peptides. It is also plausible that more open variant enzymes may be more susceptible to cleavage a nearby Arg and Lys residues, resulting in the observed doublet bands. For example, cleavage at K203 is predicted to produce a 46 kDa peptide (*) while K286 or K273 digestion would produce 36 and 38 kDa bands, respectfully (*).

bioRxiv preprint doi: https://doi.org/10.1101/2025.10.24.684457; this version posted November 19, 2025. The copyright holder for this preprint (which was not certified by peer review) is the author/funder, who has granted bioRxiv a license to display the preprint in perpetuity. It is made available under aCC-BY 4.0 International license.

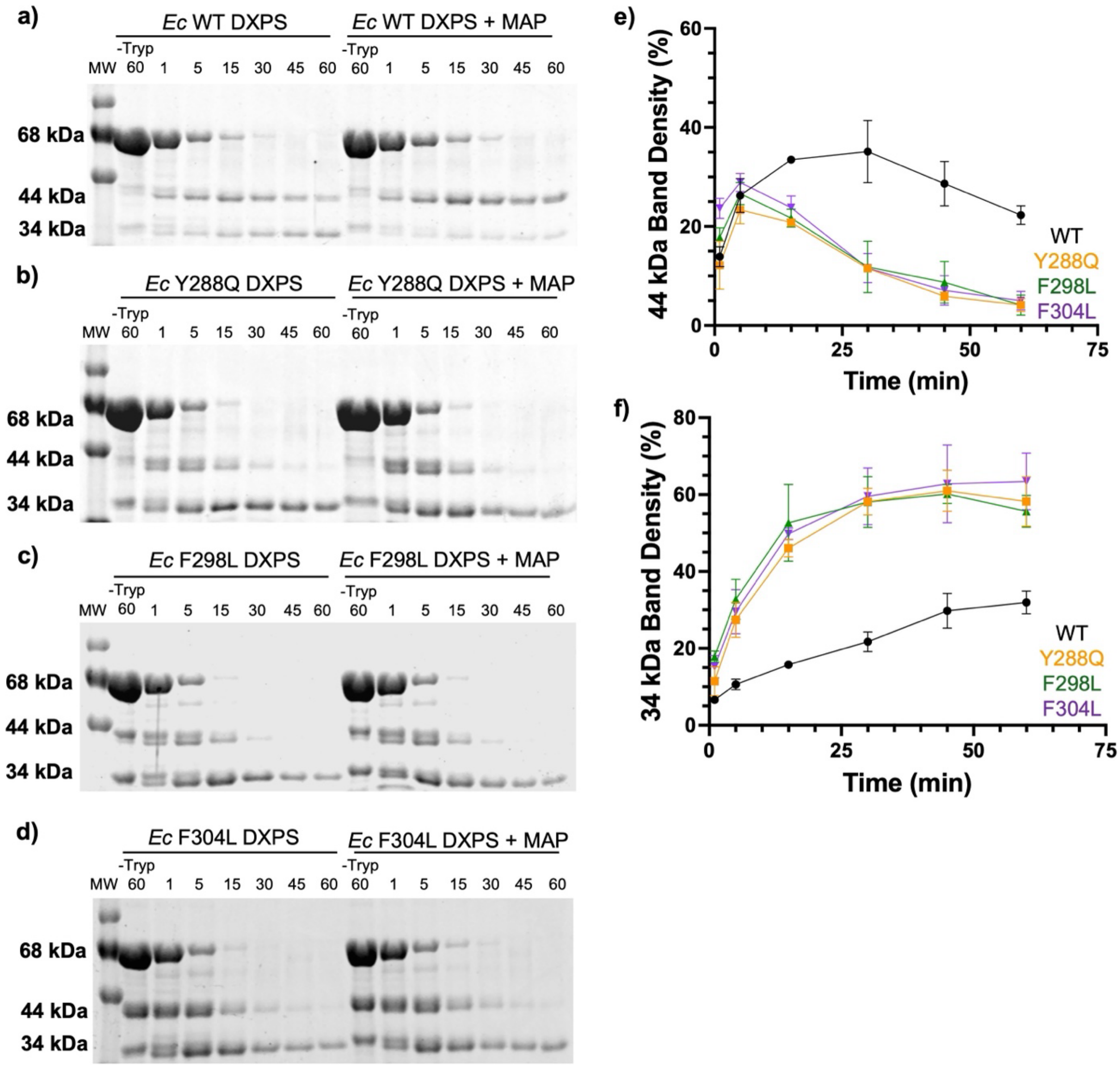

Figure S9: Limited trypsinolysis on WT and variant DXPS in the presence or absence of MAP. Representative trypsin digests are shown for (a) WT (as presented in Figure 7a), (b) Y288Q (shown in Figure 7b), (c) F298L, and (d) F304L cleavage products over 60 minutes, in the absence (left) or presence (right) of 5 mM MAP; e) Densitometry analysis of the 44 kDa and minor second band indicates this peptide accumulates to lower levels and is more rapidly converted to 33/34 kDa peptides on all DXPS variants in the absence of ligand; f) Quantification of the 34 kDa band shows rapid accumulation of all variants, relative to WT DXPS in the absence of MAP.

bioRxiv preprint doi: https://doi.org/10.1101/2025.10.24.684457; this version posted November 19, 2025. The copyright holder for this preprint (which was not certified by peer review) is the author/funder, who has granted bioRxiv a license to display the preprint in perpetuity. It is made available under aCC-BY 4.0 International license.

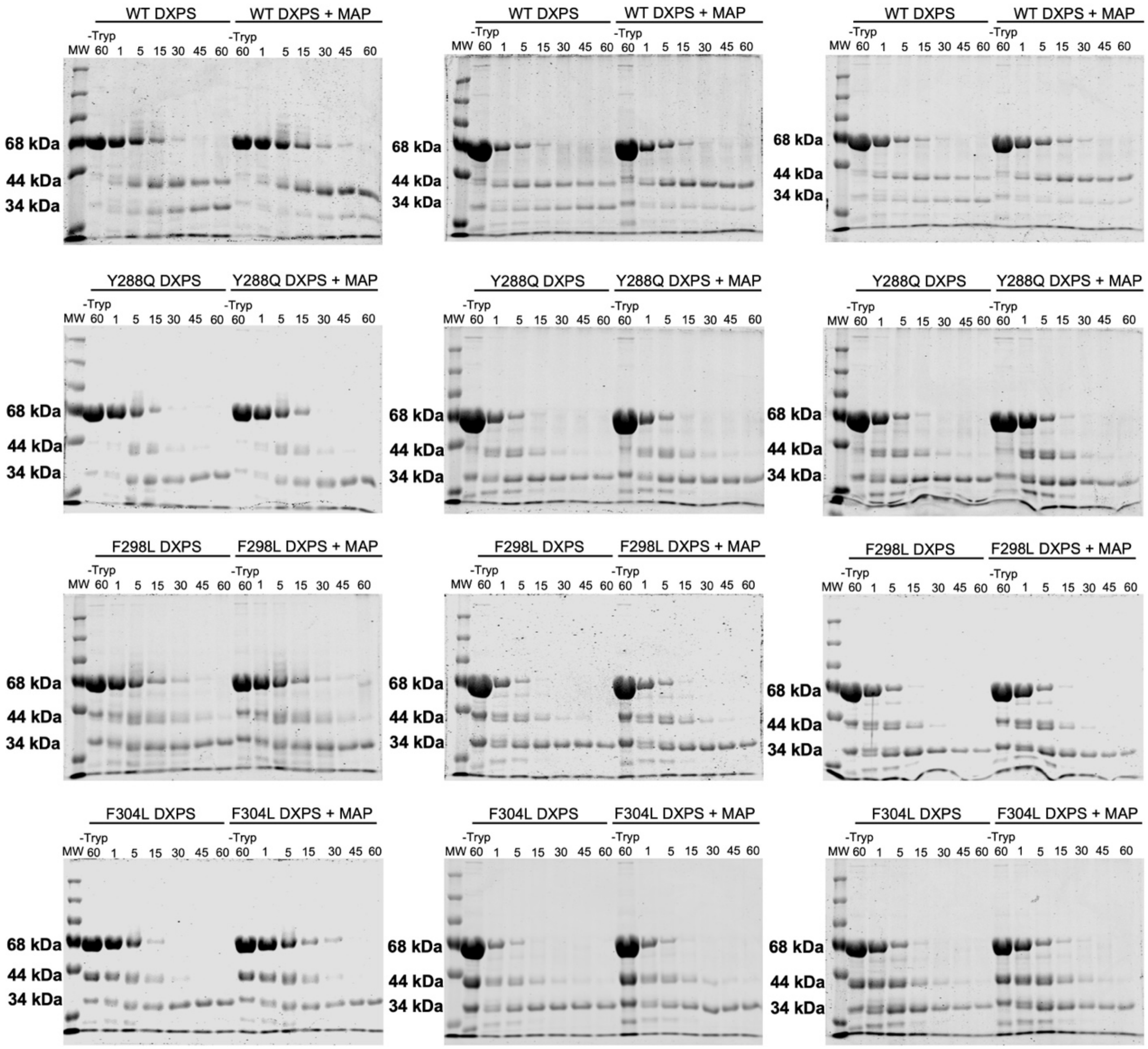

Figure S10: Gels showing replicate limited trypsinolysis experiments. Time courses of raw, unedited trypsin digests are shown for WT, Y288Q, F298L, and F304L over 60 minutes, as shown in Figures 6 and S9.

bioRxiv preprint doi: https://doi.org/10.1101/2025.10.24.684457; this version posted November 19, 2025. The copyright holder for this preprint (which was not certified by peer review) is the author/funder, who has granted bioRxiv a license to display the preprint in perpetuity. It is made available under aCC-BY 4.0 International license.

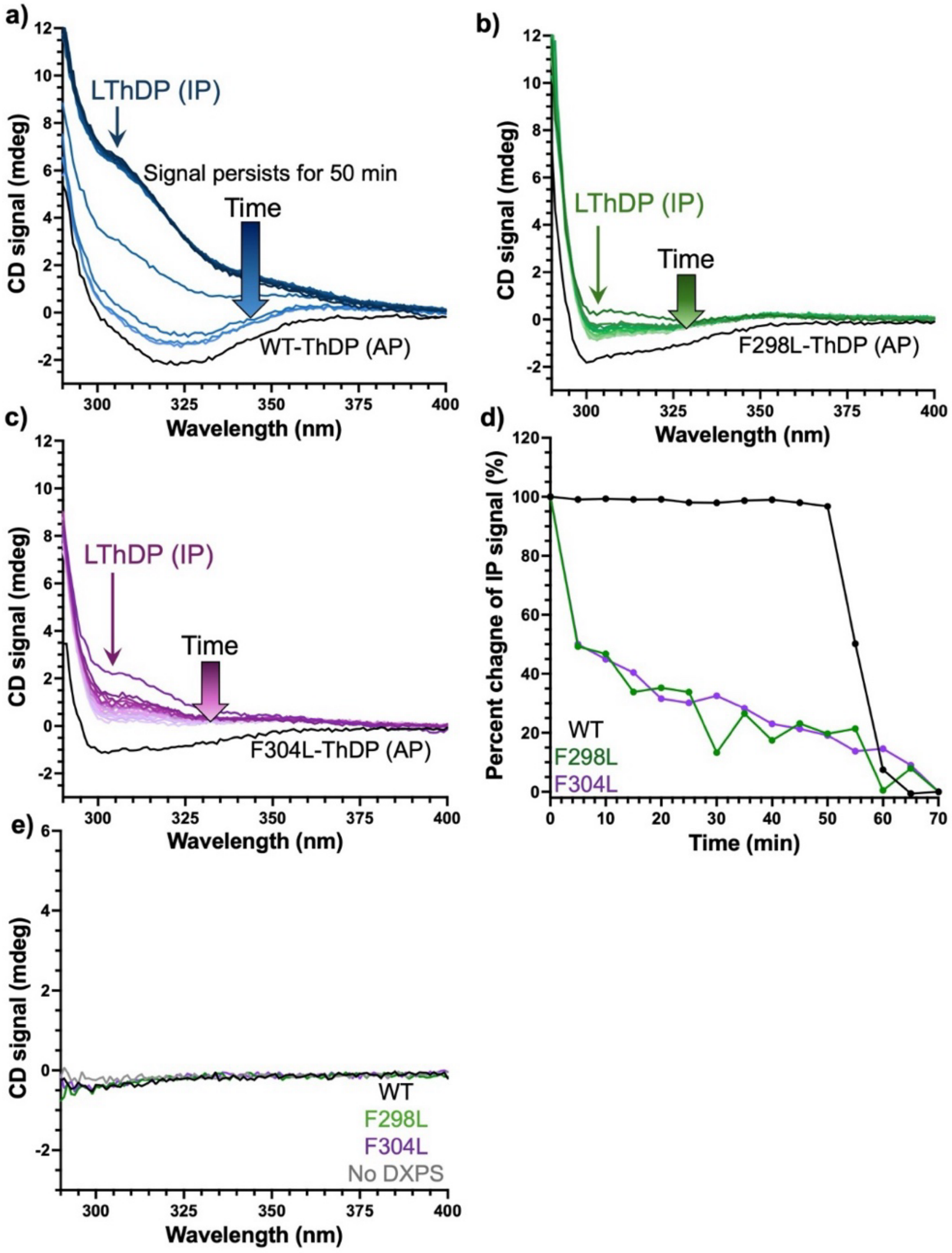

Figure S11: Duplicate CD data showing LThDP persistence (anaerobic) on (a) WT, (b) F298L, and (c) F304L DXPS. LThDP was formed upon addition of 5xK_m^{Pyr}. Intermediate was measured for 70 min where a new equilibrium observed upon signal depletion. The AP signal of each enzyme is shown in black. (d) Comparing percent change in IP signal depletion on WT, F298L, and F304L DXPS enzymes. (e) Enzyme was removed by filtration, and the clarified solution was

bioRxiv preprint doi: https://doi.org/10.1101/2025.10.24.684457; this version posted November 19, 2025. The copyright holder for this preprint (which was not certified by peer review) is the author/funder, who has granted bioRxiv a license to display the preprint in perpetuity. It is made available under aCC-BY 4.0 International license.

scanned 290-400 nm. Representative replicates in which a chiral product was not detected from pyruvate decarboxylation on WT or DXPS variants are shown. Upon depletion of the LThDP IP signal in persistence experiments, the reaction mix was returned to the anaerobic chamber where it was added to 3 kDa microfuge filters. Enzymes were filter separated by centrifugation at 14,000 x g at room temperature, and the remaining solution was added back to the 1.5 mL, 10 mm path-length cuvette (Starna cells, 1-Q-10-ST-S). A steady state CD scan was recorded of each filtrate at 25 °C from 290-400 nm with a 1 nm step and 0.5 s averaging time to determine the presence of a chiral product.

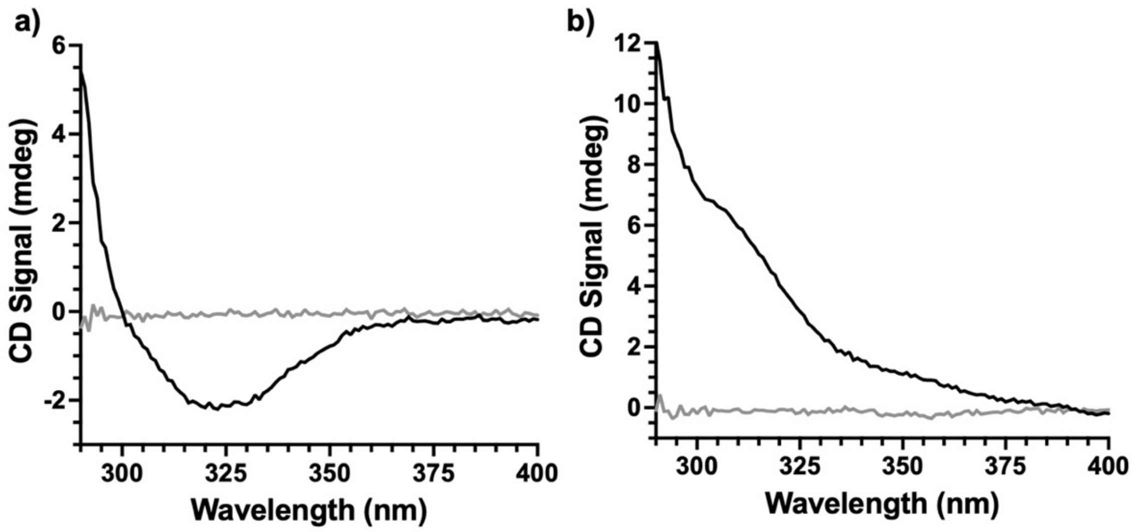

Figure S12: (a) Representative replicates comparing WT DXPS (black) and dialysis buffer (grey) alone, and (b) with $5K_m^{Pyr}$ to WT and 5mM added to the dialysis buffer sample to observe the AP and IP signatures respectfully. Refer to Figure 5a,b which first showed WT AP and IP signals.

bioRxiv preprint doi: https://doi.org/10.1101/2025.10.24.684457; this version posted November 19, 2025. The copyright holder for this preprint (which was not certified by peer review) is the author/funder, who has granted bioRxiv a license to display the preprint in perpetuity. It is made available under aCC-BY 4.0 International license.

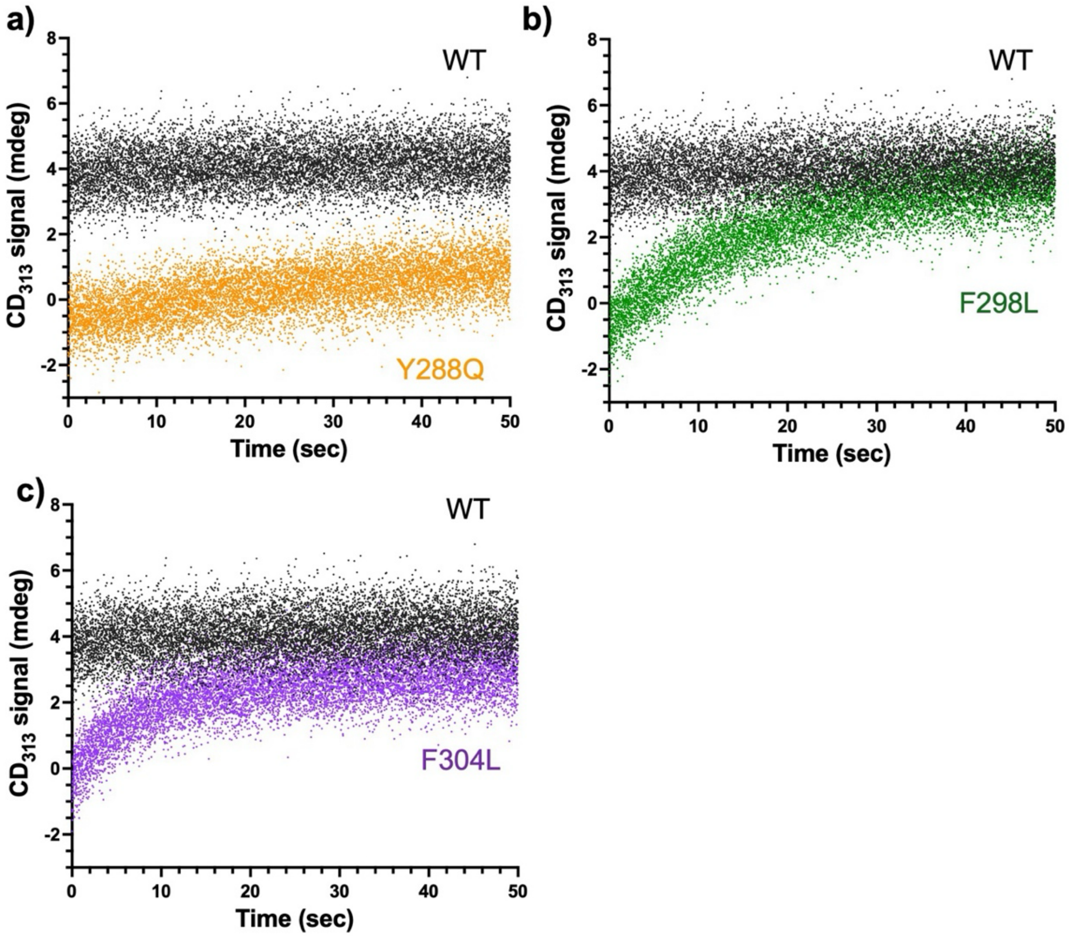

Figure S13: Disruption of aromatic cluster leads to slower PLThDP formation at 25 °C. Time-resolved experiments monitoring of the PLThDP CD_{313} signal upon addition of MAP at 25 °C revealed slower PLThDP formation when the aromatic cluster is disrupted on (a) Y288Q (orange), (b) F298L (green), and (c) F304L (purple) compared to WT DXPS (black) under conditions similar to those used to detect HCO_3^- and HEThDP by NMR. The slow intermediate formation observed at 10 °C necessitated preparation of NMR samples at ambient temperatures. Thus, we followed similar methods to compare PLThDP formation in complementary conditions to the NMR sample preparation which was used to observe the fate of pyruvate. Briefly, 60 μM WT or variant DXPS was prepared in reaction buffer containing 50 mM HEPES, pH 8, 100 mM NaCl, 2 mM $MgCl_2$, and 2 mM ThDP. A 10 mM MAP solution was prepared in a separate reaction buffer. Using the CD attached to the stopped-flow SF3 accessory, enzyme and MAP solutions (30 μM enzyme and 5 mM MAP final concentrations) were rapidly mixed. The CD_{313} signal was monitored over 50 s at 25 °C, and the data from 7 repetitive shots were averaged.

bioRxiv preprint doi: https://doi.org/10.1101/2025.10.24.684457; this version posted November 19, 2025. The copyright holder for this preprint (which was not certified by peer review) is the author/funder, who has granted bioRxiv a license to display the preprint in perpetuity. It is made available under aCC-BY 4.0 International license.

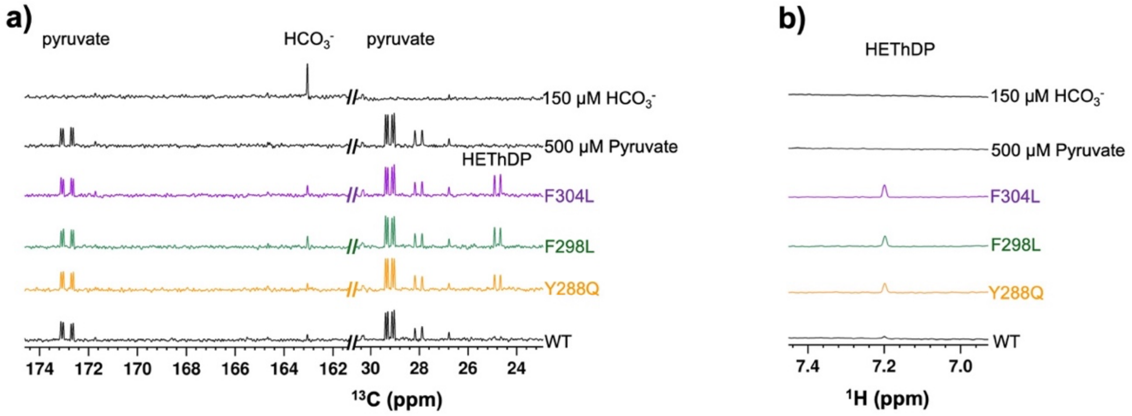

Figure S14: Observation of HEThDP ejection from Y288Q, F298L, and F304L DXPS. Duplicate data are shown of ^{13}C (a) and ^{1}H (b) spectra showing HCO_3^- and HEThDP formation upon disruption of the aromatic cluster compared to the pyruvate and HCO_3^- standards.

bioRxiv preprint doi: https://doi.org/10.1101/2025.10.24.684457; this version posted November 19, 2025. The copyright holder for this preprint (which was not certified by peer review) is the author/funder, who has granted bioRxiv a license to display the preprint in perpetuity. It is made available under aCC-BY 4.0 International license.

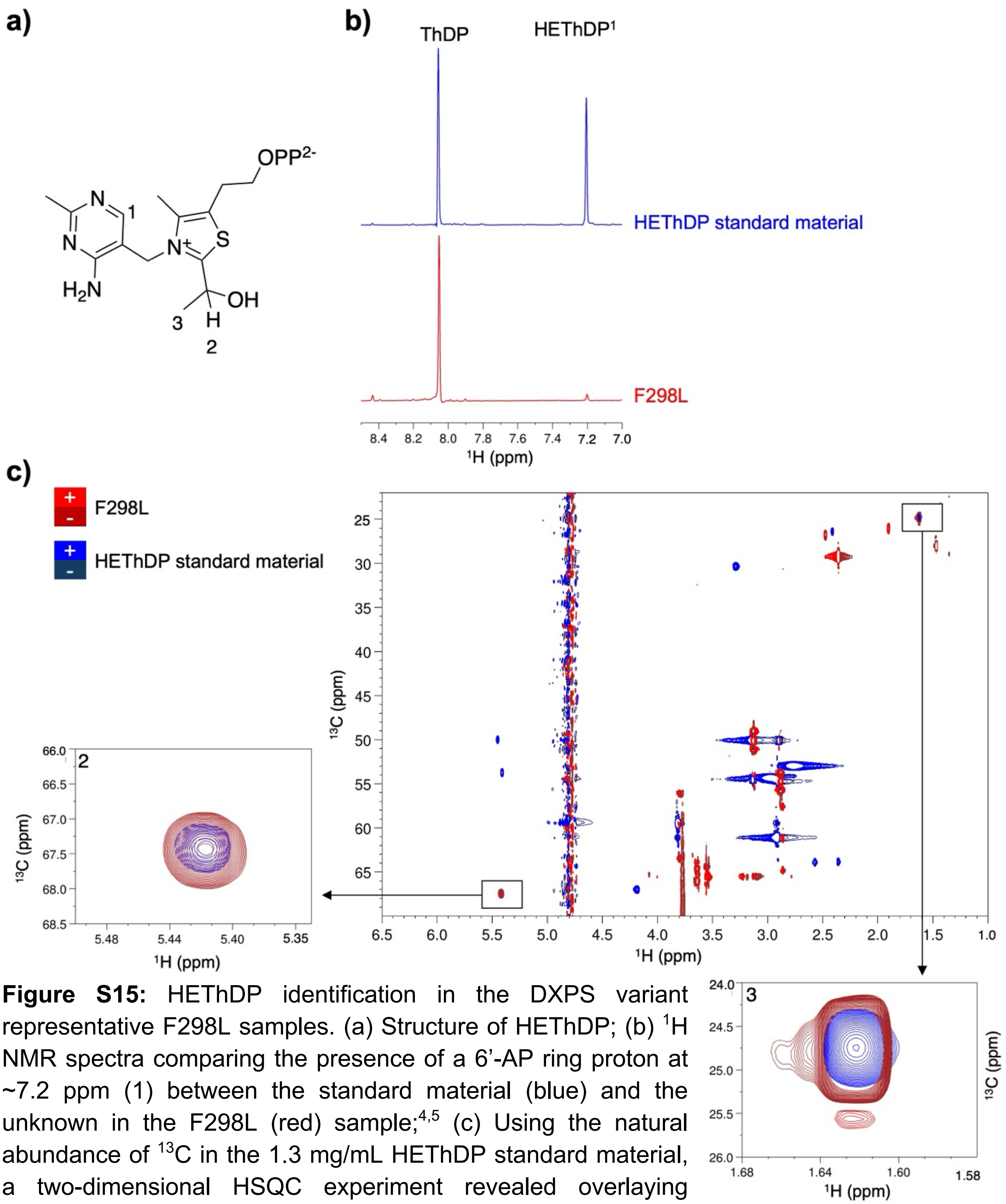

Figure S15: HEThDP identification in the DXPS variant representative F298L samples. (a) Structure of HEThDP; (b) ^{1}H NMR spectra comparing the presence of a 6'-AP ring proton at ~7.2 ppm (1) between the standard material (blue) and the unknown in the F298L (red) sample;[4,5] (c) Using the natural abundance of ^{13}C in the 1.3 mg/mL HEThDP standard material, a two-dimensional HSQC experiment revealed overlaying components of the hydroxyethyl group corresponding to $–C_{2α}$-H (2) and $-C_{2α}-CH_3$ (3) species at (5.42, 67.5) and (1.62, 24.77) ppm, respectively, between the standard and the F298L unknown sample. Together these data support HEThDP production in the F298L sample. Peaks of interest are numerically labeled on the HEThDP structure in panel a.

bioRxiv preprint doi: https://doi.org/10.1101/2025.10.24.684457; this version posted November 19, 2025. The copyright holder for this preprint (which was not certified by peer review) is the author/funder, who has granted bioRxiv a license to display the preprint in perpetuity. It is made available under aCC-BY 4.0 International license.

Two-dimensional NMR samples were prepared as described in the materials and methods for the detection of bicarbonate and HEThDP by one dimensional NMR. A HEThDP standard solution was prepared in the NMR reaction buffer at a final concentration of 1.3 mg/mL.[3] The natural abundance of ^{13}C was used to compare to the unknown product in the F298L sample chosen as a representative from the aromatic disrupting variant series. Two-dimensional NMR data was collected on a Bruker Avance spectrometer fitted with a TCI cyrogenic probe including a cyro-cooled ^{13}C preamplifier.

The HEThDP ^{1}H-^{13}C HSQC was acquired at natural abundance of ^{13}C using a gradient based experiment with the following parameters:

**Parameter**	**^{1}H**	**^{13}C**
Carrier (ppm)	4.77 (H_2O ppm @ 25 °C)	46.24
Spectral Width (ppm)	16	50
Acquisition time/FID (ms)	125	15
Complex data points	1200	114

Eight scans/FID and a recycle delay of 1.3 s were used, for a total data collection time of 45 minutes.

The F298L ^{1}H-^{13}C correlated spectrum is a modified gradient-HSQC experiment designed to (1) select for CH and CH_3 groups and eliminate CH_2 signals and (2) acquired in constant-time mode along ^{13}C so that ^{1}H-^{13}C-^{13}C signals have opposite phase relative to ^{1}H-^{13}C-^{12}C peaks. Data collection in this mode was used to confirm the presence of the hydroxyethyl $^{13}CH_3$-$^{13}C_{2a}H$-OH moiety. Data acquisition parameters are as follows:

**Parameter**	**^{1}H**	**^{13}C**
Carrier (ppm)	4.77 (H_2O ppm @ 25 °C)	46.24
Spectral Width (ppm)	14	50
Acquisition time/FID (ms)	125	15
Complex data points	1077	114

Sixteen scans/FID and a recycle delay of 1.3 s were used, for a total data collection time of 1.5 hrs.

bioRxiv preprint doi: https://doi.org/10.1101/2025.10.24.684457; this version posted November 19, 2025. The copyright holder for this preprint (which was not certified by peer review) is the author/funder, who has granted bioRxiv a license to display the preprint in perpetuity. It is made available under aCC-BY 4.0 International license.

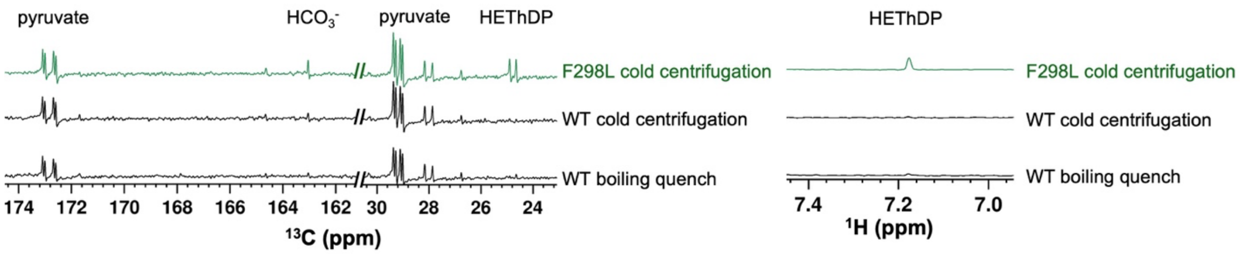

Figure S16: Discernment between pre- and post-decarboxylation intermediate by NMR ^{13}C (left) and ^{1}H (right) spectra showing accumulation of intermediate in solution in the presence of F298L DXPS when quenching by the 4 °C centrifugation method to remove enzyme using a 10 kDda filter from the sample solution. Samples were prepared in a similar manner to those described in the materials and methods to detect bicarbonate and HEThDP, with a change in the quenching method. Briefly, solutions were degassed in the anaerobic chamber, and WT or variant DXPS (5 μM) was added to NMR reaction buffer (50 mM HEPES, pH 8; 100 mM NaCl; 2 mM $MgCl_2$; 1 mM ThDP; and 5 % D_2O). Addition of 500 μM [$^{13}C_3$]pyruvate to a final reaction volume of 700 μL initiated the formation of LThDP. Samples were incubated at ambient chamber temperature (~27 °C) for 20 min. A control WT DXPS sample was quenched by boiling at 95 °C for 5 min prior to filtration using a 10 kDa microfuge filter tube by centrifugation at 14,000 x g for 10 min. A F298L DXPS sample in a filter microfuge tube was sealed with parafilm, removed from the chamber, and immediately placed on ice. The sample was centrifuged at 16,000 x g for 25 min at 4 °C in a cold room. A WT DXPS control sample was prepared as described for F298L. The filtrates in each case (600 μL) were added to prechilled microfuge tubes containing 15 μL of 10 mM gadobutrol (MedChem Express, NJ) to enhance the ^{13}C signal of HCO_3^-. The solutions were then transferred to chilled NMR tubes and stored at 4 °C until NMR acquisition which was performed the same day as sample preparation. Spectra were acquired at 150 MHz (^{13}C), at 25 °C, using a 35° ^{13}C excitation pulse, 1,320 scans/FID, 150 ms acquisition time/FID, and a 3s relaxation between scans.

bioRxiv preprint doi: https://doi.org/10.1101/2025.10.24.684457; this version posted November 19, 2025. The copyright holder for this preprint (which was not certified by peer review) is the author/funder, who has granted bioRxiv a license to display the preprint in perpetuity. It is made available under aCC-BY 4.0 International license.

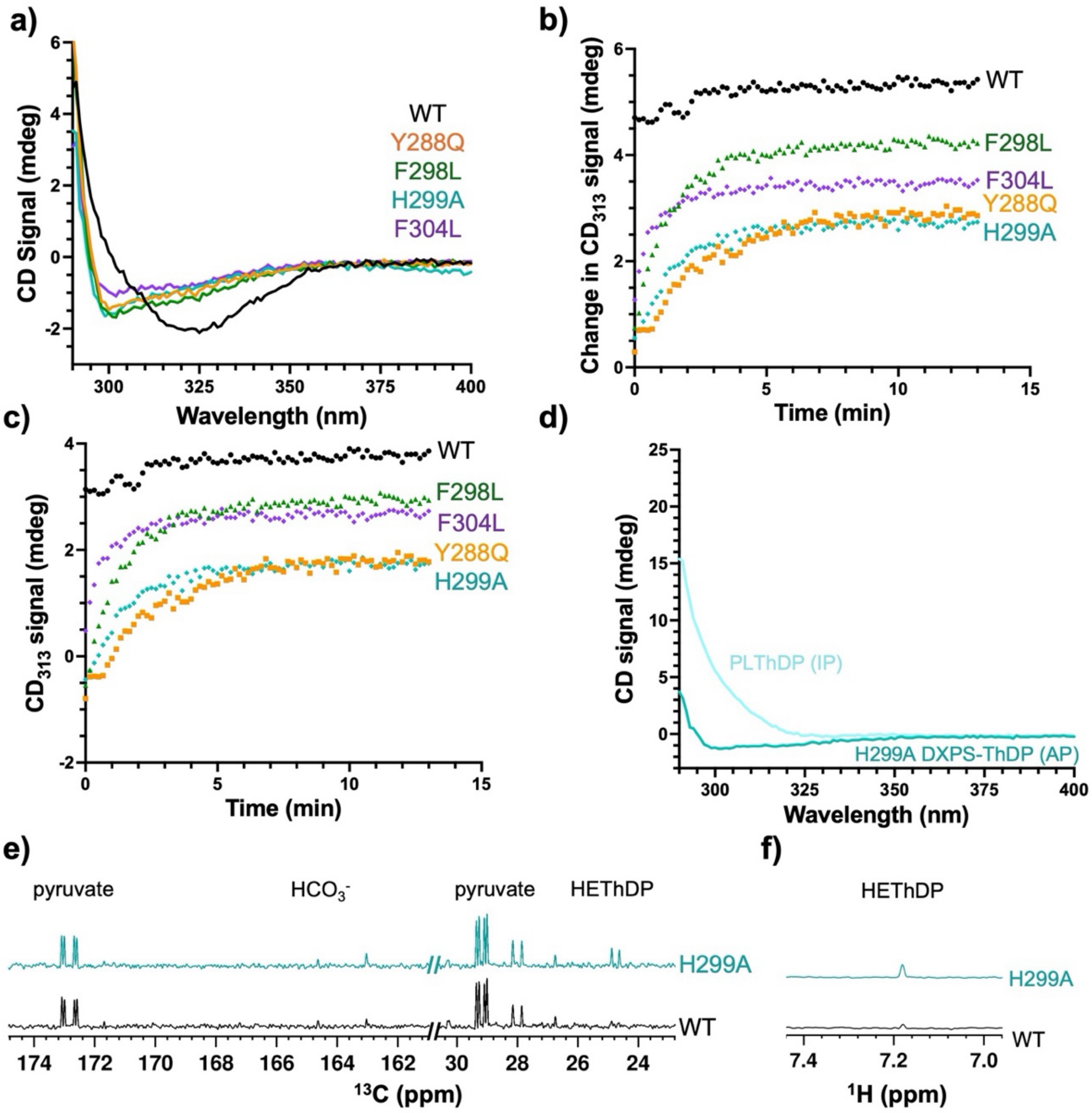

Figure S17: Duplicate data of H299A PLThDP formation overlaid with aromatic disrupting variants depicting H299A AP signal (a), normalized (b) and unnormalized (c) aerobic PLThDP formation, as shown first in figure S7, comparing WT and aromatic disrupting variant, observation of the H299A AP and PLThDP IP signals (d), ^{13}C spectra (e), and ^{1}H spectra (f).

bioRxiv preprint doi: https://doi.org/10.1101/2025.10.24.684457; this version posted November 19, 2025. The copyright holder for this preprint (which was not certified by peer review) is the author/funder, who has granted bioRxiv a license to display the preprint in perpetuity. It is made available under aCC-BY 4.0 International license.

References

(1) Chen, P. Y.-T.; DeColli, A. A.; Freel Meyers, C. L.; Drennan, C. L. X-Ray Crystallography-Based Structural Elucidation of Enzyme-Bound Intermediates along the 1-Deoxy-d-Xylulose 5-Phosphate Synthase Reaction Coordinate. *J. Biol. Chem.* **2019**, *294* (33), 12405–12414.
(2) DeColli, A. A.; Zhang, X.; Heflin, K. L.; Jordan, F.; Freel Meyers, C. L. Active Site Histidines Link Conformational Dynamics with Catalysis on Anti-Infective Target 1-Deoxy-d-Xylulose 5-Phosphate Synthase. *Biochemistry* **2019**, *58* (49), 4970–4982.
(3) Gruys, K. J.; Halkides, C. J.; Frey, P. A. Synthesis and Properties of 2-Acetylthiamin Pyrophosphate: An Enzymatic Reaction Intermediate. *Biochemistry* **1987**, *26* (24), 7575–7585.
(4) Tittmann, K.; Golbik, R.; Uhlemann, K.; Khailova, L.; Schneider, G.; Patel, M.; Jordan, F.; Chipman, D. M.; Duggleby, R. G.; Hübner, G. NMR Analysis of Covalent Intermediates in Thiamin Diphosphate Enzymes. *Biochemistry* **2003**, *42* (26), 7885–7891.
(5) Ullrich, J.; Mannschreck, A. Studies on the Properties of (–)-2-Alpha-Hydroxyethyl-Thiamine Pyrophosphate (“active Acetaldehyde”). *Eur. J. Biochem.* **1967**, *1* (1), 110–116.
